# Supplementary material for: Functional role of 18 LysR-Type transcriptional regulators of Salmonella enterica serovar Typhi
Source: PLoS One. 2025 Dec 5;20(12):e0338130. doi: 10.1371/journal.pone.0338130 (PMC12680141; doi:10.1371/journal.pone.0338130)
Supplement: S1 Table — (DOCX) [file pone.0338130.s001.docx]

**S1 Table. Bacterial strains and plasmids used in this work.**

| **Strains** | **Genotype and/or relevant characteristics** | **Reference** |
| --- | --- | --- |
| IMSS-1 | *Salmonella enterica* serovar Typhi 9.12, d, serotype; Mexican reference clinical strain | (10) |
| ∆STY0036 | IMSS-1 ∆STY0036 | (7) |
| ∆STY0159 | IMSS-1 ∆STY0159 | (58) |
| ∆STY0277 | IMSS-1 ∆STY0277 | (58) |
| ∆STY0341 | IMSS-1 ∆STY0341 | (58) |
| ∆STY0651 | IMSS-1 ∆STY0651 | (58) |
| ∆STY0730 | IMSS-1 ∆STY0730 | (58) |
| ∆STY1537 | IMSS-1 ∆STY1537 | This study |
| ∆STY1578 | IMSS-1 ∆STY1578 | This study |
| ∆STY1693 | IMSS-1 ∆STY1693 | (58) |
| ∆STY2510 | IMSS-1 ∆STY2510 | (58) |
| ∆STY2660 | IMSS-1 ∆STY2660 | (58) |
| ∆STY2821 | IMSS-1 ∆STY2821 | (58) |
| ∆STY3158 | IMSS-1 ∆STY3158 | (58) |
| ∆STY3165 | IMSS-1 ∆STY3165 | This study |
| ∆STY3415 | IMSS-1 ∆STY3415 | (58) |
| ∆STY3547 | IMSS-1 ∆STY3547 | (58) |
| ∆STY4196 | IMSS-1 ∆STY4196 | (58) |
| ∆STY4468 | IMSS-1 ∆STY4468 | (58) |
| *E. coli* DH5α | Φ80d/*lac*Z∆M15 ∆(*lac*ZYA-*arg*F) U169 *rec*A1*end*A1 *hsd*R17 (r_k_^-^m_k_^+^) *pho*A*sup*E44 λ-thi-*1 gyr*A96 *rel*A. Nal^R^ | Gibco BRL |
| **Plasmids** |  |  |
| pCP20 | FLP^+^, λ cI857^+^, λ PR Rep^ts^, AP^R^, Cm^R^ | (15) |
| pKK232-8 | pBR322 derivative containing a promoterless chloramphenicol acetyltransferase (*cat*) gene, Ap^R^. | Pharmacia LKB Biotechnology |
| pKK232-9 | pKK232-8 derivative containing a promoterless chloramphenicol acetyltransferase (*cat*) gene, Km^R^. | (6) |
| pKK232-9-STY0036*-*471+90 | pKK232-9 containing 471 bp upstream and 90 bp downstream of the STY0036 ATG start codon, Km^R^. | This study |
| pKK232-9-STY0159*-*265+84 | pKK232-9 containing 265 bp upstream and 84 bp downstream of the STY0159 ATG start codon, Km^R^. | This study |
| pKK232-9-STY0277*-*346+129 | pKK232-9 containing 346 bp upstream and 129 bp downstream of the STY2660 ATG start codon, Km^R^. | This study |
| pKK232-9-STY0341*-*332+178 | pKK232-9 containing 332 bp upstream and 178 bp downstream of the STY0341 ATG start codon, Km^R^. | This study |
| pKK232-9-STY0651*-*376+100 | pKK232-9 containing 376 bp upstream and 100 bp downstream of the STY0651 ATG start codon, Km^R^. | This study |
| pKK232-9-STY0730*-*320+89 | pKK232-9 containing 320 bp upstream and 89 bp downstream of the STY20730 ATG start codon, Km^R^ | This study |
| pKK232-9-STY1537*-*382+143 | pKK232-9 containing 382 bp upstream and 143 bp downstream of the STY1537 ATG start codon, Km^R^. | This study |
| pKK232-9-STY1578*-*363+97 | pKK232-9 containing 363 bp upstream and 97 bp downstream of the STY1578 ATG start codon, Km^R^. | This study |
| pKK232-9-STY1693*-*383+129 | pKK232-9 containing 383 bp upstream and 129 bp downstream of the STY1693 ATG start codon, Km^R^. | This study |
| pKK232-9-STY2510*-*374+94 | pKK232-9 containing 374 bp upstream and 94 bp downstream of the STY2510 ATG start codon, Km^R^. | This study |
| pKK232-9-STY2660*-*395+118 | pKK232-9 containing 395 bp upstream and 118 bp downstream of the STY2660 ATG start codon, Km^R^. | This study |
| pKK232-9-STY2821*-*371+93 | pKK232-9 containing 371 bp upstream and 93 bp downstream of the STY2821 ATG start codon, Km^R^. | This study |
| pKK232-9-STY3158*-*403+192 | pKK232-9 containing 403 bp upstream and 192 bp downstream of the STY3158 ATG start codon, Km^R^. | This study |
| pKK232-9-STY3165*-*476+117 | pKK232-9 containing 476 bp upstream and 117 bp downstream of the STY3165 ATG start codon, Km^R^. | This study |
| pKK232-9-STY3415*-*345+99 | pKK232-9 containing 345 bp upstream and 99 bp downstream of the STY3415 ATG start codon, Km^R^. | This study |
| pKK232-9-STY3547*-*393+185 | pKK232-9 containing 393 bp upstream and 185 bp downstream of the STY3547 ATG start codon, Km^R^. | This study |
| pKK232-9-STY4196*-*700+115 | pKK232-9 containing 700 bp upstream and 115 bp downstream of the STY4196 ATG start codon, Km^R^. | This study |
| pKK232-9-STY4468*-*365+103 | pKK232-9 containing 365 bp upstream and 103 bp downstream of the STY4468 ATG start codon, Km^R^. | This study |
