# Supplementary material for: Functional role of 18 LysR-Type transcriptional regulators of Salmonella enterica serovar Typhi
Source: PLoS One. 2025 Dec 5;20(12):e0338130. doi: 10.1371/journal.pone.0338130 (PMC12680141; doi:10.1371/journal.pone.0338130)
Supplement: S2 Table — (DOCX) [file pone.0338130.s002.docx]

**S2 Table. Oligonucleotides used in this work for mutants construction and transcriptional fusions.**

| **Oligonucleotides** | **Sequence (5´-3´)** |
| --- | --- |
| STY1537-H1P1 | TCACGAATTGAGAAAACCATGGACCTGACGCAACTGGAAATGTGTAGGCTGGAGCTGCTTCG |
| STY1537-H2P2 | TCGCAGGCCGTTTCGTCTTCAGTCGGCGCTCGCCAGTTGGGCCATATGAATATCCTCCTTAG |
| STY1578-H1P1 | AAATAAGACGTTAAAAATATGAATATCGAATTACGTCATTTATGTAGGCTGGAGCTGCTTCG |
| STY1578-H2P2 | CACCCTTTTTAAGCGTAATTAATCCTGTCGGGCAGCCAGTAACATATGAATATCCTCCTTAG |
| STY3165-H1P1 | CGATAAAGTAGATAGGTTATGCGTTACTCACCTGAAGCGTTATGTAGGCTGGAGCTGCTTCG |
| STY3165-H2P2 | ATTTGACGGTGGCGCACGTTATTTCTGCTGTAAATGGTAGCGCATATGAATATCCTCCTTAG |
| STY0036-471-450BamHI-F | CGGGATCCACGCATTAGCGTCACCTTTAGC |
| STY0036+90+68KpnI-R | GGGGTACCACGCATTAGCGTCACCTTTAGC |
| STY0159-265-241BamHI-F | CGGGATCCGGTATAAGGGCCATACATATCTGC |
| STY0159+84+59KpnI-R | GGGGTACCGTGAAGCTATGGTTTTCAGCAATC |
| STY0277-346-322BamHI-F | CGGGATCCCCGCCGTAAACAAGACCATCAGC |
| STY0277+129+106KpnI-R | GGGGTACCATCTCCAGCTTTTTAACTGCCC |
| STY0341-332-308BamHI-F | CGGGATCCGATACAATTAATGCGCTCCAGAC |
| STY0341+178+154KpnI-R | GGGGTACCGATGTGAATCGCGCCGAAGTAAAG |
| STY0651-376-357BamHI-F | CGGGATCCAACAGGTTGTTGGTTTAC |
| STY0651+100+76KpnI-R | GGGGTACCGTGTTTCCGCTGCTTTGCTAATGC |
| STY0730-320-296BamHI-F | CGGGATCCGCAACAGCATCATCACGACCATC |
| STY0730+89+64KpnI-R | GGGGTACCGAAACGCCAATATCAAGATCCTGTG |
| STY1537-382-359BamHI-F | GGGGATCCGGATTTCGTCACTTCCGCACGC |
| STY1537+143+121KpnI-R | GGGGTACCAACAGAGAGACGCCCAGTTCGG |
| STY1578-363-340BamHI-F | CGGGATCCGGTAGAAATAGAAAGTGAAACG |
| STY1578+97+75KpnI-R | GGGGTACCGCGGCTGCGAGATATTGAGGCG |
| STY1693-383-360BamHI-F | CGGGATCCCCACAGCATCCCCAGACTTCCG |
| STY1693+129+107KpnI-R | GGGGTACCCCACTCCTCTAACTGACGAACG |
| STY2510-374-352BamHI-F | CGGGATCCATATTTTCCCACCCACTGCCCG |
| STY2510+94+71KpnI-R | GGGGTACCGGCGGATTGCGTCATGTATAGC |
| STY2660-395-373BamHI-F | CGGGATCCAAAGCCGCTGTAGAGCATAGGG |
| STY2660+118+96KpnI-R | GGGGTACCCTTTTACGCTATGACTGACCGC |
| STY2821-371-349BglII | GAAGATCTCTAAAGAAAACAGGATACCGGC |
| STY2821+93+71KpnI-R | GGGGTACCTGATGGCTGAGTGATAAATAGG |
| STY3158-403-381BamHI-F | CGGGATCCATCAGGGCACGTTTGCGGATC |
| STY3158+192+170KpnI-R | GGGGTACCCTTGCACCGTCGGATGTAAG |
| STY3165-476-454BamHI-F | CGGGATCCGCAAAAAGAACAGGAAGAAACC |
| STY3165+117+94KpnI-R | GGGGTACCGCAATGGCGGTACTAATGGTGG |
| STY3415-345-322BamHI-F | CGGGATCCTGCCGTCGCTGTCGCGGTATTCC |
| STY3415+99+75KpnI-R | GGGGTACCGCCCCAACTCATCAGCCGCTGC |
| STY3547-393-371BamHI-F | CGGGATCCCTTCAACCTCAAACGAACAGGC |
| STY3547+185+163KpnI-R | GGGGTACCCCAGCTTCGGTGAGCCCAATGC |
| STY4196-700-680BamHI-F | CGGGATCCGAATGTGCTGCTGCAAACTG |
| STY4196+115+93KpnI-R | GGGGTACCGTATTTGCCGTGAGACGCTGCC |
| STY4468-365-345BamHI-F | CGGGATCCACGACGGCGGGAACAAAG |
| STY4468+103+80MluI-R | CGACGCGTCTTGCTAATGGTGGGCTGGGTG |

BamHI, BglII, KpnI and MluI restriction site are underlined.
