# Supplementary material for: Functional role of 18 LysR-Type transcriptional regulators of Salmonella enterica serovar Typhi
Source: PLoS One. 2025 Dec 5;20(12):e0338130. doi: 10.1371/journal.pone.0338130 (PMC12680141; doi:10.1371/journal.pone.0338130)
Supplement: S3 Table — (DOCX) [file pone.0338130.s003.docx]

**S3 Table. List of proteins and models used for structural alignment.**

| **Protein/Model ID** | **Structure ID (PDB or AlphaFold)** | **Source** | **Reference** |
| --- | --- | --- | --- |
| TsaR | 3fxu_A | PDB | (16) |
| CrgA | 3hhg_A | PDB | (17) |
| ArgP | 3isp_A | PDB | (18) |
| BenM | 3k1m_A | PDB | (19) |
| AphB | 3szp_A | PDB | (20) |
| OxyR | 4x6g_A | PDB | (21) |
| VV2_1132 | 5y9s_A | PDB | (22) |
| HypT | HypT | PDB | (23) |
| HinK | 6m5f_A | PDB | (24) |
| STY2660 | AF-A0A3Y9LZ30-F1 | AlphaFold |  |
| STY3547 | AF-Q8XFH1-F1 | AlphaFold |  |
| STY3158 | AF-Q8XGD5-F1 | AlphaFold |  |
| STY4468 | AF-Q8Z1R4-F1 | AlphaFold |  |
| STY4196 | AF-Q8Z281-F1 | AlphaFold |  |
| STY3415 | AF-Q8Z3L0-F1 | AlphaFold |  |
| STY3165 | AF-Q8Z3Z7-F1 | AlphaFold |  |
| STY2821 | AF-Q8Z4K8-F1 | AlphaFold |  |
| STY2510 | AF-Q8Z557-F1 | AlphaFold |  |
| STY1693 | AF-Q8Z6P0-F1 | AlphaFold |  |
| STY1578 | AF-Q8Z6X7-F1 | AlphaFold |  |
| STY1537 | AF-Q8Z703-F1 | AlphaFold |  |
| STY0730 | AF-Q8Z8F4-F1 | AlphaFold |  |
| STY0651 | AF-Q8Z8K2-F1 | AlphaFold |  |
| STY0341 | AF-Q8Z942-F1 | AlphaFold |  |
| STY0277 | AF-Q8Z987-F1 | AlphaFold |  |
| STY0159 | AF-Q8Z9G0-F1 | AlphaFold |  |
| STY0036 | AF-Q8Z9P3-F1 | AlphaFold |  |
