# Supplementary material for: Functional role of 18 LysR-Type transcriptional regulators of Salmonella enterica serovar Typhi
Source: PLoS One. 2025 Dec 5;20(12):e0338130. doi: 10.1371/journal.pone.0338130 (PMC12680141; doi:10.1371/journal.pone.0338130)
Supplement: Attachment — Submitted filename: Rviewer coments.docx [file pone.0338130.s005.docx]

**Peer Review of Manuscript PONE-D-25-53096**

This manuscript presents a highly comprehensive and systematic functional characterization of 18 previously uncharacterized LysR-Type Transcriptional Regulators (LTTRs) in *Salmonella enterica* serovar Typhi.

**Overall Assessment**

**Major Revisions** are suggested to enhance the mechanistic depth and structural completeness of the manuscript.

**Major Scientific and Structural Suggestions**

**1. Mechanistic Validation: Genetic Complementation is Strongly Recommended**

While the authors utilized the technically sound method of non-polar mutagenesis to minimize transcriptional polar effects, it is standard practice in molecular genetics to provide definitive proof of cause-and-effect. Therefore, it is strongly recommended that genetic complementation be performed for all LTTR deletion mutants exhibiting a significant phenotype.

**2. Target Gene Validation for Key Regulators**

To advance the paper from a phenotypic screen to a mechanistic study, evidence connecting the LTTR to its transcriptional targets would be valuable. I suggest quantifying the expression of at least one strong **candidate downstream target gene** using RT-qPCR. This would provide a direct molecular link between the LTTR and the pathway it controls.

**3. Complete Main Text Integration of Remaining Results**

While the data for the final experiments are present in the some Figures, the main text draft appears structurally incomplete. The narrative and discussion for the following results should be fully written and integrated into the main body of the manuscript:

- LTTR regulation of **outer membrane proteins (OMPs)** (corresponding to Figure 7).
- The results of the **high temperature resistance assays** (corresponding to Figure 8).

**4. Integration of Structural and Phenotypic Data**

The structural classification of the 18 LTTRs is a novel and informative starting point. Explicitly discuss the relationship between the LTTR structural groupings (HinK-like, AphB-like, etc.) and their observed physiological phenotypes. It would be insightful to discuss whether structural similarity correlates with functional redundancy, or to offer a rationale for any observed functional divergence in *S. Typhi*.

**Minor Clarity and Presentation Suggestions**

1. **Control Data Documentation:** The important control data demonstrating that the ΔLTTR mutants show no change in basal growth kinetics compared to the wild-type strain in N-MM ("Data no shown") **should be included** in a **Supplementary Figure** or **Supplementary Table** to fully document the experimental controls.
2. **CAT Assay Precision:** For reproducibility, please clarify the cell harvest stage for the CAT assays in the Methods section. Instead of the current wording, specifying a standardized measurement (e.g., "mid-log phase, OD595≈0.6") would be preferred.
3. **Keywords:** Please ensure that all acronyms used as Keywords are spelled out in full upon their first mention in the text (e.g., "N-minimal medium (N-MM)").
