# Supplementary material for: Functional role of 18 LysR-Type transcriptional regulators of Salmonella enterica serovar Typhi
Source: PLoS One. 2025 Dec 5;20(12):e0338130. doi: 10.1371/journal.pone.0338130 (PMC12680141; doi:10.1371/journal.pone.0338130)
Supplement: Attachment — Submitted filename: Response to Reviewers.docx [file pone.0338130.s006.docx]

**Peer Review of Manuscript PONE-D-25-53096**

This manuscript presents a highly comprehensive and systematic functional characterization of 18 previously uncharacterized LysR-Type Transcriptional Regulators (LTTRs) in *Salmonella enterica* serovar Typhi.

**Overall Assessment**

**Major Revisions** are suggested to enhance the mechanistic depth and structural completeness of the manuscript.

**Major Scientific and Structural Suggestions**

**Responses to reviewers are in bold**.

**1. Mechanistic Validation: Genetic Complementation is Strongly Recommended**

While the authors utilized the technically sound method of non-polar mutagenesis to minimize transcriptional polar effects, it is standard practice in molecular genetics to provide definitive proof of cause-and-effect. Therefore, it is strongly recommended that genetic complementation be performed for all LTTR deletion mutants exhibiting a significant phenotype.

**Response: I agree with your suggestions and in this regard, we previously show that deletions in one of the LTTRs presented here (STY0036), shows diverse phenotypes such as sensibility to bile salts and deoxycholic acid as well as the absence of OmpC and OmpF. Complementation experiments with the promoter region and with the entire STY0036 gene reestablish the growth in bile salts, deoxycholic acid as well as the porin profile. This result shows that the mutants generated in this work have not polar effects. However future studies on individual LTTRs clearly need complementation experiments as well as their involvement in *S*. Typhi regulatory networks.**

**2. Target Gene Validation for Key Regulators**

To advance the paper from a phenotypic screen to a mechanistic study, evidence connecting the LTTR to its transcriptional targets would be valuable. I suggest quantifying the expression of at least one strong candidate downstream target gene using RT-qPCR. This would provide a direct molecular link between the LTTR and the pathway it controls.

**Response: I appreciate your observation and a strong candidate for the regulation for one of the LTTR described here (STY2660) was STY2661. Hence LTTR STY2660 (WP_001109834.1) was selected for the study that you suggest, since is widely conserved in the *Salmonella* genus and is divergent to a bile acid: sodium symporter STY2661, indicating that STY2660 have a role in the resistance to bile salts mediated by STY2661. However only STY2660 was involved in bile and deoxycholic acid resistance and STY2661 was not involved in bile acids and in deoxycholic acid resistance, despite being a bile acid: sodium symporter (10). Thus, sometimes LTTRs regulate their divergent genes, however in the case mentioned above STY2660 was unable to regulate it divergent STY2661 gene.**

**However, data generated by our group shows that the LTTRs STY2660 and STY0036 directly control the expression of the master porin regulator *ompR,* and this protein interact and induces the expression of both *ompC* and *ompF*. Thus, this LTTRs are pivotal indirectly in the *ompC* and *ompF* genetic expression in *S*. Typhi (8, 10). This paragraph was included in the new version of the manuscript.**

**Furthermore, we previously demonstrate that LTTRs are able to regulate genes located elsewhere in the genome (Hernandez Lucas et al 2018). Thus, the involvement of the regulatory network of each specific LTTR described here is a matter of future studies.**

**3. Complete Main Text Integration of Remaining Results**

While the data for the final experiments are present in some Figures, the main text draft appears structurally incomplete. The narrative and discussion for the following results should be fully written and integrated into the main body of the manuscript:

- LTTR regulation of **outer membrane proteins (OMPs)** (corresponding to Figure 7).
- The results of the **high temperature resistance assays** (corresponding to Figure 8).

**Response: Corrected as suggested**

**4. Integration of Structural and Phenotypic Data**

The structural classification of the 18 LTTRs is a novel and informative starting point. Explicitly discuss the relationship between the LTTR structural groupings (HinK-like, AphB-like, etc.) and their observed physiological phenotypes. It would be insightful to discuss whether structural similarity correlates with functional redundancy, or to offer a rationale for any observed functional divergence in *S. Typhi*.

**Response:** **In this respect the structural data generated in this work shows the presence of four structural LTTR groups, and the literature of some of them such as STY0036, STY2660, CrgA, AphB and OxyR demonstrated that these LTTRs are involved in motility, biofilm formation, porin regulation and bile salts resistance (8,10,39,45,51).**

**Based on these data we decided evaluated the mentioned phenotypes, and additional human stress conditions faced by S. Typhi in the infection process, as well as inducing conditions of Salmonella pathogenic islands I and II of the 18 LTTRs described here.**

**Minor Clarity and Presentation Suggestions**

1. **Control Data Documentation:** The important control data demonstrating that the ΔLTTR mutants show no change in basal growth kinetics compared to the wild-type strain in N-MM ("Data no shown") **should be included** in a **Supplementary Figure** or **Supplementary Table** to fully document the experimental controls.

**Response:** Corrected as you suggested

2. **CAT Assay Precision:** For reproducibility, please clarify the cell harvest stage for the CAT assays in the Methods section. Instead of the current wording, specifying a standardized measurement (e.g., "mid-log phase, OD595 ≈0.6") would be preferred. **Response:** Corrected as you suggested
3. **Keywords:** Please ensure that all acronyms used as Keywords are spelled out in full upon their first mention in the text (e.g., "N-minimal medium (N-MM)").

**Response:** **Corrected as you suggested**

**Major Scientific and Structural Suggestions**

**Responses to reviewers are in bold**.

Reviewer #1: This manuscript presents a highly comprehensive and systematic functional characterization of 18 previously uncharacterized LysR-Type Transcriptional Regulators (LTTRs) in *Salmonella enterica* serovar Typhi. The high-throughput approach, covering key virulence-associated phenotypes such as bile tolerance, antimicrobial resistance, motility, and biofilm formation, represents a significant contribution to *S.* Typhi pathogenesis research. The methodological rigor, particularly the use of non-polar mutagenesis and host-relevant minimal medium, makes this work suitable for publication in PLOS ONE, pending revisions.

Overall Assessment:

Major Revisions are suggested to enhance the mechanistic depth and structural completeness of the manuscript.

Reviewer #2: 1. The authors should clarify whether the identified LTTRs directly regulate ompC and ompF transcription or whether their effects are indirect. Likewise, for biofilm formation and motility, it would strengthen the manuscript to indicate whether specific virulence- or flagellar-related genes are misregulated in the mutants. Including even preliminary data or a focused discussion on potential regulatory targets would greatly enhance the study’s impact.

**Response:**

**Regarding the first point about porin regulation, previous work by our group has focused on the *ompC* and *ompF* regulation mediated by LTTRs, and the results demonstrate that the LTTRs STY2660 and STY0036 described in this work, directly control the expression of the master porin regulator *ompR* and this protein directly induces the expression of both *ompC* and *ompF*. Thus, this LTTRs are pivotal indirectly in the *ompC* and *ompF* genetic expression in *S*. Typhi (7, 9). This paragraph was included in the new version of the manuscript.**

**Regarding the second point we perform a string analysis of the 18 LTTRs described in this work and the results shows that** **the LTTR STY0341 characterized in this study is homologous to SinR from *Salmonella* Typhimurium. Previous studies have shown that *sinR* mutants are attenuated in their ability to replicate within macrophages, and that *sinR* expression is regulated by HilD, a major transcriptional activator involved in *Salmonella* invasion of intestinal epithelial cells (58,59). These findings suggest that STY0341 could also play a role in *Salmonella* Typhi virulence. Furthermore, *in silico* analysis using STRING (60) revealed that STY0341 is co-expressed with HypT, a protein involved in oxidative stress resistance in *S.* Typhimurium. In contrast, the STRING analysis of the remaining 17 LTTRs showed no co-expression with known virulence genes or with transcriptional regulators associated with Salmonella's free-living state. This paragraph was included in the new version of the manuscript.**

2. The assignment of mutants into four functional groups appears somewhat arbitrary and is not consistently aligned with the experimental data. For example, the manuscript states that Group II mutants “produced OmpF but not OmpC,” yet the corresponding figures suggest that some Group II strains express both porins, while others like ΔSTY0341 (also classified as Group II) lack OmpF entirely. The authors should carefully re-evaluate this classification using all available phenotypic evidence and provide a clear, data-driven justification for the final groupings.

**Response:**

**As you suggest a carefully revision of the section mentioned was performed and a mistake in group III regarding motility establish that motility was affected negatively. However, the correct phenotype is that motility was enhanced. The new revised version of the manuscript includes the correction.**

3. The introduction would benefit from a brief mention of the clinical significance of typhoid fever and the growing challenge of antibiotic resistance. This context would more effectively underscore the relevance of investigating virulence mechanisms in Salmonella typhi.

**Response:**

**We included a brief mention of the clinical significance of typhoid fever and the growing challenge of antibiotic resistance in the introduction as you suggested.**

4. In the discussion, the authors should either temper or remove the claim regarding the global regulon of the transcription factor, as 1D gel electrophoresis alone is insufficient to support such a broad conclusion. A comprehensive assessment of a regulon would require more robust approaches, such as proteomic or transcriptomic analyses.

**Response:**

**We are in agreement with your observation; thus we eliminated this paragraph of the discussion as you suggested.**
